# Editorial

**Published:** 1891-09

**Authors:** 


### Editorial.

### EXAMINING BOARD.

We understand that a bill will be introduced into the Georgia Legislature looking to the establishment of an Examining Board in this State, and though we have not had an opportunity to examine the text of the law, yet we trust that in the end some such legislation will pass.

Though there are many features of the boards that we think could be modified, it is generally accepted that they have done good wherever they have had a fair opportunity for work. It is an unfortunate fact that every year many graduates are sent out who are not prepared to enter upon the practice of medicine, and there is no way to reach such cases except through the examining power that is not connected with the institution granting the diploma. This gives the field for the work of the boards, and where they do not fix a standard that is unreasonable, they are of great benefit, and do not work injustice. We trust that the physicians in the legislature will in some way arrange this bill so that the board can have in its composition some of those who, by college connection, or otherwise, have some experience in examining of classes, for we are fully convinced that such is a very important item. We are sure that the entire profession of the State will be glad to see a well equipped and effective Examining Board.

### DR. HOLMES' SANITARIUM.

Dr. J. B. S. Holmes, of Rome, Georgia, will have in a month's time, or by the first of October, his Sanitarium at that place ready for the reception of patients. The doctor's long experience with the treatment of diseases peculiar to women, has convinced him of the importance of a private hospital for the successful management of many conditions that are every day baffling the skill of our most experienced physicians, who are compelled to undertake their management at the homes of patients. Dr. Holmes is an accomplished gynecologist, and richly deserves the extended reputation he enjoys. Born and reared under the sunny clime of Georgia, he possesses all the congeniality characteristic of a Southern gentleman. We bespeak for him a bright future and a position on the top round of the ladder of fame and distinction.

The Sanitarium has about one hundred rooms, is well finished, thoroughly heated, well ventilated, with perfect sanitary arrangements. The maternity and surgical division will be made as near aseptic as possible. The rooms all have open fire-places, have inside and outside blinds, gas and electric bells, are all nicely carpeted and handsomely furnished; in short, the institution will be strictly first-class in all of its appointments, even to the minutest detail. The building is supplied with a splendid hydraulic passenger elevator, and nothing will be left undone that will add in any degree to the comfort of patients. The grounds consist of about five acres, the lawn is large and handsome—altogether a lovely home. See his advertisement on another page.

### SUBSTITUTION.

There has been much said of late in the lay press upon the subject of substitution by druggists, attention having been called to some of its phases by Mr. A. Frank Richardson, in his address before the National Editorial Association. The form of substitution that is more especially mentioned is that of proprietary medicines that have by merit achieved success. It seems that there are a number of manufacturing houses in this country whose sole work is to make articles that, as closely as the law permits, counterfeit the genuine, and in many cases the name of the dispensing druggist is put upon the label. When a customer calls for the article which has been imitated, he is told that there is none of it on hand, but he can be served with one equally as effective, "and of our own make," which is in many cases cheaper at the same time. It is said that one article alone is imitated more than two hundred times. It is nothing more than right that anything that has, by its merit, attained to a large degree of success, should be permitted to enjoy the same, and such underhanded methods should be prevented by law. In the meantime, the consumer who wants a certain article should see that he secures the original package, protected as it is by the law of copyright.

MESSRS. J. B. LIPPINCOTT COMPANY announce that they will publish, about September 1st, the eighth edition of Wood's Therapeutics: its Principles and Practice; rearranged, rewritten, and enlarged. Scarcely three years have elapsed since the appearance of the seventh edition, yet the preparation of the present volume has necessitated a careful study by its author of more than seven hundred memoirs. In the present edition no revolutionary changes have been made comparable to those of the seventh revision, but great care has been exercised to see that every portion of the work has been thoroughly revised, and a number of the articles have been completely rewritten, while some new drugs have been noticed. Among those portions of the book which are practically new may be mentioned, as important, the whole subject of Anæsthetics, the articles on Cocaine, Strophanthus, Caffeine, Antipyrin, Antifebrin, Phenacetin, Hydrastine, Paraldehyd, Lead-poisoning, etc. Among the absolutely new articles may be mentioned Sulphonal, Chloralamid, Aristol, and others.

---

### AMERICAN GYNECOLOGICAL SOCIETY.

The sixteenth annual meeting of this association will be held in the lecture room of the Columbian University, corner 15th and H streets, Washington, D. C., on Sept. 22d, 23d and 24th, with the following officers: President, A. Reeves Jackson, Chicago; Vice-Presidents, J. T. Johnson, Washington; W. H. Baker, Boston; Secretary, Henry C. Coe, New York.

The programme shows that there will be a great many valuable papers read.

---

The Mississippi Valley Medical Association will hold its seventeenth annual session at St. Louis, Wednesday, Thursday and Friday, October 14, 15 and 16, 1891. A large attendance, a valuable program and a good time are expected. The members of the medical profession are respectfully invited to attend. E. S. MCKEE, M. D., Sec'y.

*57 West Seventh St., Cincinnati.*

### TRI-STATE MEDICAL ASSOCIATION.

The third annual meeting of the Tri-State Medical Association will convene in Turner Hall, Chattanooga, Tennessee, Tuesday, October 27th, 1891, and continue in session three days. Indications are that it will be one of the largest medical meetings ever held in the South. Representative physicians from all sections will be present.

All who desire to read papers should send title to the Secretary of the Association before September 1st. In due time a circular will be issued giving a complete list of all papers and names of exhibitors who apply for space before October 1st.

W. L. GAHAGAN, Sec'y of Executive Com.
P. O. Box 542, Chattanooga, Tenn.

---

MEMBERSHIP IN THE AMERICAN MEDICAL ASSOCIATION.—This is obtainable, at any time, by a member of any State or local medical society which is entitled to send delegates to the Association. All that is necessary is for the applicant to write to the Treasurer of the Association, Dr. Richard J. Dungliso Lock Box 1274, Philadelphia, Pa., sending him a certificate or statement that he is in good standing in his own society, signed by the President and Secretary of said society, with five dollars for annual dues. Attendance as a delegate at an annual meeting of the Association is not necessary in order to obtain membership. On receipt of the above amount the weekly Journal of the Association will be forwarded regularly.

---

We wish to call special attention to our advertisements.

